# Social communication and emotion difficulties and second to fourth digit ratio in a large community-based sample

**DOI:** 10.1186/s13229-015-0063-7

**Published:** 2015-12-28

**Authors:** Manuela Barona, Radha Kothari, David Skuse, Nadia Micali

**Affiliations:** Institute of Child Health, 30 Guilford Street, London, WC1N 1EH UK; Department of Psychiatry, Icahn Medical School at Mount Sinai; Mindich ChildHealth and Development Institute, Icahn Medical School at Mount Sinai, 31428 Madison Ave, New York, NY 10029 United States

**Keywords:** ALSPAC, Social communication, Emotion recognition difficulties, Second to fourth digit ratio, Testosterone, Autism

## Abstract

**Background:**

Recent research investigating the extreme male brain theory of autism spectrum disorders (ASD) has drawn attention to the possibility that autistic type social difficulties may be associated with high prenatal testosterone exposure. This study aims to investigate the association between social communication and emotion recognition difficulties and second to fourth digit ratio (2D:4D) and circulating maternal testosterone during pregnancy in a large community-based cohort: the Avon Longitudinal Study of Parents and Children (ALSPAC). A secondary aim is to investigate possible gender differences in the associations.

**Methods:**

Data on social communication (Social and Communication Disorders Checklist, *N* = 7165), emotion recognition (emotional triangles, *N* = 5844 and diagnostics analysis of non-verbal accuracy, *N* = 7488) and 2D:4D (second to fourth digit ratio, *N* = 7159) were collected in childhood and early adolescence from questionnaires and face-to-face assessments. Complete data was available on 3515 children. Maternal circulating testosterone during pregnancy was available in a subsample of 89 children.

**Results:**

Males had lower 2D:4D ratios than females [*t* (3513) = −9.775, *p* < 0.001]. An association was found between measures of social communication and emotion recognition, and the lowest 10 % of 2D:4D ratios. A significant association was found between maternal circulating testosterone and left hand 2D:4D [OR = 1.65, 95 % CI 1.1–2.4, *p* < 0.01].

**Conclusions:**

Previous findings on the association between 2D:4D and social communication difficulties were not confirmed. A novel association between an extreme measure of 2D:4D in males suggests threshold effects and warrants replication.

**Electronic supplementary material:**

The online version of this article (doi:10.1186/s13229-015-0063-7) contains supplementary material, which is available to authorized users.

## Background

There has been a growing interest in studying the effects of prenatal testosterone exposure on later developmental outcomes both in animal [1, 2] and human studies [3–5] in the last decades. Evidence from animal studies has shown that variations in prenatal testosterone exposure can lead to gender differences in behavior, cognition, and brain structure [6, 7]. Furthermore, recent studies have investigated this association using amniotic androgen levels, with results suggesting that prenatal testosterone exposure might be associated with individual differences in cognitive development [8–10]. However, research studying the relationship between normal prenatal variability in testosterone and postnatal behavior in humans is challenging. Due to the high economic costs and health risks associated with direct measurement, prenatal testosterone is generally assessed through the use of proxy measures, such as second to fourth digit ratio and maternal circulating testosterone levels during pregnancy. Here, we aim to investigate the association between social communication and emotion recognition difficulties and prenatal testosterone exposure using indirect measures.

Research studying the higher prevalence of autism spectrum disorders (ASD) in males (4:1, male to female) has led to the hypothesis that the masculinizing effect of fetal testosterone may play a role in the development of ASD [11]. This hypothesis is based on the extreme male brain (EMB) theory of autism, which is part of the broader empathizing vs. systemizing (E/S) theory of gender differences in cognitive styles in healthy subjects [12, 13]. According to this theory, the main difference between male and female brains is that whilst the former is driven to analyze variables in the system to derive a rule (systemize), the latter is driven to identify another person’s emotions and responds in an appropriate manner (empathize). Although we all have empathizing and systemizing skills, individual variation gives rise to different brain types, with females relying more on empathizing and males more on systemizing. Baron-Cohen and colleagues argued that the bias towards males in ASD could be understood as an extreme manifestation of the physiological and psychological characteristics of the male brain [8]. This theory aims to explain the higher prevalence of ASD in males, and based on this, we would expect that children with ASD in general would have higher testosterone.

The ratio between the index (2D) and ring (4D) finger (2D:4D) is the most frequently used indirect approach to measure fetal testosterone exposure. However, the association between prenatal testosterone and 2D:4D has been a controversial one [14, 15]. 2D:4D has been shown to be sexually dimorphic (males on average have lower 2D:4D than women) [16, 17] and correlates negatively with fetal testosterone/estradiol ratio (i.e., high levels of fetal testosterone/fetal estradiol ratios are associated with low 2D:4D) [18]. Furthermore, an experimental study by Zheng and Cohn in 2011 demonstrated the influence of prenatal testosterone and prenatal estradiol on the development of 2D:4D in the mouse [19]. Another proxy measure that has been used in the past is maternal circulating testosterone during pregnancy. Testosterone is liposoluble and can therefore cross the placenta; bidirectional transfer of androgens between mother and fetus is thus theoretically plausible, although placental aromatase likely converts most testosterone into estrogen. However, some studies have shown that maternal testosterone during pregnancy is correlated with fetal testosterone in typical mothers [20–22]. There are also controversies about the effect of maternal testosterone during pregnancy on gender role behavior of preschool children [3, 23, 24]. Evidence from studies on women with hyperandrogenemia in pregnancy suggests that maternal testosterone may affect fetal testosterone levels only in female fetuses. However, studies have shown that maternal testosterone levels are not higher in women carrying male fetuses [25, 26].

Support for an association between 2D:4D and ASD comes from reports demonstrating that children with ASD, and their first-degree relatives, have lower 2D:4D (hypermasculinised) ratios than population normative values [27]. Findings in the literature have been inconsistent [28–32] however, this might be due to the differences in populations studied and differences in the way finger ratio was measured and used. Two meta-analyses [33, 34] found that on average, individuals with ASD tend to have lower digit ratios compared to control groups, independent of type of finger measurement, with effect sizes of *d* = −0.58 [34] and *d* = −0.43 [33].

A large body of research has also focused on investigating the association between systemizing and empathizing and prenatal testosterone in both clinical and population samples, with inconsistent findings [35–39]. In a large community-based study, Manning and colleagues, found evidence of a negative correlation between systemizing and 2D:4D in adults [35]. A recent meta-analysis [34] showed no association and small effect sizes between systemizing/empathizing and 2D:4D in healthy adults. Furthermore, despite heterogeneous findings, this review found that heterogeneity in the findings did not moderate the overall effect of the findings.

Studies have also examined the association between 2D:4D and the Autism Quotient Test (AQ), which measures levels of ASD-like traits in the general population; however, as with previous studies, findings are mixed and inconclusive [38–41]; as summarized in a meta-analysis by Teatero and Netley in 2013 [33]. More specific investigations have been conducted investigating the association between 2D:4D and ASD-like traits, such as central coherence, social cognition and interaction, and communication skills [38, 41].

The review by Teatero and Netley in 2013 [33] reported that although individuals with ASD tend to have lower digit ratios and results tend to be consistent with the EMB theory, the great variability in these findings highlights the need for studies to further investigate the association between fetal testosterone and ASD-like traits. Furthermore, they underlined the need to conduct studies with large enough samples to provide adequate power, and to consider the effects of gender given that sex differences in measures, such as the AQ, may be contributing to associations with 2D:4D.

Hence, we aimed to firstly investigate the association between 2D:4D and social communication and emotion recognition in a large community-based sample, and secondly, to test the correlation between measures of maternal circulating testosterone levels during pregnancy and 2D:4D in a subset. Based on previous findings of an association between high prenatal testosterone exposure and autism/autistic-like traits, we predicted that low 2D:4D would be associated with increased social communication and emotion recognition difficulties. More specifically, based on the EMB theory of autism, we predicted that extreme low 2D:4D ratio in females (which correspond to a high prenatal testosterone) would be associated with increased social communication and emotion recognition difficulties. We also predicted an association between maternal circulating testosterone and finger ratios, based on previous findings.

## Methods

### Participants

The Avon Longitudinal Study of Parents and Children (ALSPAC) is a longitudinal population study investigating the environmental and genetic factors that affect health and development. All pregnant women, in a predefined study area of Avon, whose expected date of delivery was between April 1, 1991 and December 31, 1992, were eligible. Initially, 14,541 women, and the children they were expecting, were enrolled in the study [42, 43]. Children and parents have been followed up for the last 21 years. Behavioral assessments were conducted through clinics. Parental consent was obtained on behalf of all study participants under 16 years of age. Please note that the study website contains details of all the data that is available through a fully searchable data dictionary [http://www.bris.ac.uk/alspac/researchers/data-access/data-dictionary/].

### Exposures

#### 2D:4D ratio

At 11 years of age, the children had their hands photocopied, and the length of their second and forth digits were measured using digital calipers. 2D:4D ratio was calculated by dividing the length of the second digit by the length of the fourth digit. A random subsample of children’s hands was measured in vivo to determine the validity of using photocopies to measure digit length ratio. The analysis yielded a high correlation (*r* > 0.97) between photocopies and in vivo measures for both hands. From the ALSPAC sample, 7159 children attended the clinic when the 2D:4D measure was carried out.

### Maternal testosterone levels during pregnancy

Maternal blood samples were obtained by venipuncture during routine prenatal medical care in a subsample of the overall ALSPAC cohort (*N* = 89). Samples were taken based on the timing of women’s medical appointments (raging between 5 and 36 weeks gestation; mean: week 16 and SD: 8 weeks). Time of day when blood was sampled was not fixed. Once obtained, blood was spun to form plasma aliquots of 5 ml. Assays of testosterone were conducted by the Lewis Laboratories, Southmead Hospital, U.K. Testosterone was measured using an automated chemi-luminescence system (SCS) from Cheron Diagnostics. The assay shows high specificity for T and the assays range is 0.35 to 52.0 nmol/L. The intra-assay and inter-assay coefficients of variation are 11.3 and 13.8 % at 1.7 nmol/L and 4.9 and 7.7 % at 43.8 nmol/L. For details regarding the assays see Hines et al. [3].

### Outcomes

#### Facial emotion recognition: DANVA (at 8.5 years)

The Diagnostic Analysis of Nonverbal Accuracy (DANVA) [44] was designed to measure individual differences in the accurate sending and receiving of non-verbal social information. The receptive facial expressions subtest used in this study measures the child’s ability to recognize an emotion from facial cues. Participants were shown photographs of children expressing one of the four basic emotions: happiness, sadness, anger, or fear and two different levels of emotion expression intensity: high and low. This leads to a total of ten outcomes measuring different emotions as well as intensities and misattributions, based on evidence from the literature suggesting that deficits in emotion recognition vary depending on emotion and intensity. Higher scores in this task represent more errors or misattributions when recognizing the emotion. Ten binary scores indicating whether children made more (above cut-off) or less (below cut-off) errors/misattributions were used. DANVA scores were subject to floor effects which lead to the positive skew in the data, therefore, cut-offs for each of the variables were derived in collaboration with the author who developed the DANVA (Stephen Nowicki) for ALSPAC and were based upon the distribution of results in the whole sample (see Kothari et al. [45]). This specific cut-offs have been previously used in ALSPAC papers [46, 47]. The overall construct validity of the DANVA was examined in a sample of 1001 children age 6 through 10 years old [44]. Evidence for validity was strong; Cronbach’s alphas for the receptive tests ranged from 0.77 to 0.88, and an overall test-retest reliability of 0.84. From the ALSPAC sample, 7488 children attended the clinic when the DANVA was conducted.

### Social communication: SCDC (at 13.5 years)

The Social and Communication Disorders Checklist [48] is a 12-item questionnaire to be completed by parents about their children’s social interaction and communication skills. A higher SCDC score reflects more difficulties. Research has shown that the measure has an excellent internal consistency (0.93), high test-retest reliability (0.81), and high heritability in both genders (0.74) [48]. Additionally, when using a score of 9 or above, the SCDC has been found to be predictive of autism [49]. This cut-off score was determined in the ALSPAC sample by Skuse and colleagues in 2009 [49].

### Emotion recognition from social cues: the emotional triangles task [50] (at 13.5 years)

This test involves the use of computerized abstract animations to measure the participant’s ability to attribute an emotional mental state to non-human animate entities. The animations are used to test the participant’s ability to use motion cues, such as speed and trajectory of movement, and movement in relation to others, to infer emotions. Participants are shown 5-s animations of a circle and a triangle on a computer screen. In some of the animations, the triangle moves in a self-propelled manner designed to evoke a particular emotion: angry, happy, sad, or scared. In the other animations, it moves in a manner designed to make it appear “non-living.” Participants are asked (a) whether the triangle is living, and if so, how living (measured on a Likert scale 0–5), or (b) whether the triangle has a particular emotion (happy, sad, angry, or scared). For more details on the task, scoring or animations, see Boraston et al. [50]. Four outcome variables representing each of the four emotions assessed were used, with a higher score representing better emotion recognition ability. Of the whole ALSPAC sample, 5844 children attended the clinic when the emotional triangles task was conducted.

Children were eligible for this study if they had completed: the emotion recognition tasks, had a valid 2D:4D measure, and if their parents had returned the Social Communication Disorders Checklist (SCDC). The final sample of children with data on all four measures was 3515.

### Data analyses

All variables were examined individually to check for inconsistencies/outliers and normality. For participants with less than 25 % missing data on the SCDC, total scores were calculated using prorating [51].

Scores for both DANVA and SCDC were not normally distributed. Scores obtained from the DANVA were positively skewed. The measure is scored by simply adding up the number of errors/misattributions for each emotion, and since a significant number of children made few or no errors, DANVA scores were subject to floor effects which lead to the positive skew in the data. Binary scores from the DANVA were derived in collaboration with the author for the measure and used as per Kothari et al. [45].The SCDC score was used as a binary variable using a cut-off of ≥9 which has been found to be predictive of a diagnosis of autism [49]. This binary variable was preferred to count variables, so that results would be relevant to a clinical ASD population. Scores from the emotional triangles task were normally distributed.

2D:4D was used as a continuous variable, and the ratios for both left and right hands were computed by multiplying each individual score by 100 (e.g., 0.97 × 100 = 97) in order to reduce the number of decimal points. A binary variable was also calculated using a cut-off of the lowest 10 % ratios (i.e., to index the most male-biased ratio). This was calculated separately for both sexes given gender differences.

Firstly, gender differences were investigated for all measures using *t* test and chi-square analyses. The association between 2D:4D and social communication/emotion recognition was explored using regression analyses (logistic regression for the DANVA and SCDC and linear regression for the emotional triangles task). Scores for DANVA, SCDC, and emotional triangles were included as outcome variables in separate analyses, and 2D:4D ratio was included as the predictor. Correlations between maternal testosterone levels and 2D:4D ratio was explored using Spearman’s correlations in the subsample with data on maternal testosterone.

Regression analyses were conducted, using initially a minimally adjusted model (model 1) where gender, age at completion, and tester were adjusted for as a priori confounders. Additional confounders (ethnic background, mother’s age, maternal education, and parity) were adjusted for in a second fully adjusted model (model 2). Further analyses were run stratified by gender. Post hoc regression analyses were also run using an extreme cut-off (bottom 10 %, equal to extreme levels of prenatal testosterone) of the 2D:4D.

All analyses were run using SPSS 21 (SPSS Inc., USA), the Bonferroni-Holm method was used to adjust the significance level for multiple testing [52].

### Attrition and missingness

Attrition and missingness was predicted by sociodemographic factors. The overall missingness was predicted by child gender, child ethnicity, parity, marital status of the mother, and maternal education. (Additional file 1: Table S1).

Multiple random imputation was used to deal with missing covariate data in the covariates (which ranged between 1.1 and 2.2 %), and both predictors and outcomes were used in the model. Missing data was imputed for maternal education, parity, child’s ethnicity, and relationship stability. Results for both complete and imputed cases were almost identical; consequently, the results based on the imputed cases are presented here as multiple imputation is assumed to correct bias.

### Procedure

The ALSPAC Law and Ethics Committee and Local Research Ethics Committees approved the study. Full information was provided to the participants, and consent was acquired before any questionnaires were sent or assessments carried out.

## Results

### Sociodemographic data

Results are shown in Additional file 2: Table S2. Sociodemographic characteristics were studied across the sample.

### Gender differences

As expected from past literature, there was a significant difference between male and female’s 2D:4D ratios [*t* (3513) = *−*9.775, *p* < 0.001, with males having a lower 2D:4D than females. Distributions and gender differences for all measures are in Additional file 3: Table S3.

### Digit ratio and social communication/emotion recognition

After adjusting for multiple testing, no significant associations were found between 2D:4D ratios (entered as a continuous variable) and DANVA scores (see Table 1). These findings did not change the following stratification by gender (see Table 2). No significant associations were found between 2D:4D ratios (entered as a continuous variable) and scores in SCDC after adjusting for multiple testing (see Table 3). These findings did not change following stratification by gender (see Table 3). No significant associations were found between 2D:4D ratios (entered as a continuous variable) and scores in emotional triangles after adjusting for multiple testing (see Table 4). These findings did not change following stratification by gender (see Table 4). In other words, there was no association between 2D:4D ratios and autistic traits, whether measured by ratings of behavior (SCDC) or by two distinctly different measures of emotion recognition (DANVA and emotional triangles).

**Table 1:** Logistic regression analyses of children’s 2D:4D ratios: comparison of children scoring high and low on emotion recognition (DANVA)

	Minimally adjusted model OR (95 % CI)^a^		Fully adjusted model OR (95 % CI)^b^	
	2D:4D right	2D:4D left	2D:4D right	2D:4D left
DANVA				
Happy faces (≥1 error)	1.01 (0.95, 1.03)	0.99 (0.97, 1.02)	1.01 (0.99, 1.03)	0.99 (0.97, 1.02)
Sad faces (≥2 errors)	1.00 (0.98, 1.03)	1.03 (1.00, 1.05)	1.00 (0.98, 1.03)	1.03 (1.00, 1.05)
Angry faces (≥4 errors)	1.01 (0.99, 1.04)	1.02 (0.98, 1.05)	1.02 (0.99, 1.04)	1.02 (0.99, 1.05)
Fearful faces (≥3 errors)	0.99 (0.97, 1.03)	0.99 (0.96, 1.02)	1.00 (0.97, 1.03)	0.99 (0.97, 1.02)
All low intensity faces (≥5 errors)	1.03 (1.00, 1.06)^ns^	1.01 (0.99, 1.04)	1.03 (1.00, 1.06)^ns^	1.02 (0.99, 1.04)
All high intensity faces (≥3 errors)	1.01 (0.98, 1.03)	1.00 (0.97, 1.02)	1.01 (0.98, 1.03)	1.00 (0.97, 1.02)
Misattributed as Happy (≥4)	0.99 (0.96, 1.02)	1.00 (0.97, 1.03)	0.99 (0.96, 1.02)	1.00 (0.97, 1.03)
Misattributed as sad (≥ three)	1.01 (0.98, 1.04)	1.00 (0.96, 1.02)	1.01 (0.98, 1.04)	0.99 (0.96, 1.02)
Misattributed as Angry (≥2)	1.01 (0.98, 1.05)	1.01 (0.98, 1.04)	1.01 (0.98, 1.05)	1.01 (0.98, 1.04)
Misattributed as Fearful (≥2)	1.00 (0.98, 1.03)	1.00 (0.97, 1.02)	1.00 (0.98, 1.03)	1.00 (0.97, 1.02)

Higher scores on SCDC represent more social communication problems. Lower scores in DANVA means a poorer performance in identifying emotions/more misattributions

*ns* not significant after adjusting for multiple comparisons using the Bonferroni-Holm method

^a^Minimally adjusted model: adjusted for child age, child gender, and tester

^b^Fully adjusted model: adjusted for child age, child gender, tester, maternal education, ethnic background and parity

**Table 2:** Logistic regression analyses children’s 2D:4D ratios stratified by gender: comparison of children scoring high and low on emotion recognition (DANVA)

	Minimally adjusted model OR (95 % CI)^a^		Fully adjusted model OR (95 % CI)^b^	
	2D:4D right	2D:4D left	2D:4D right	2D:4D left
Female				
Happy faces (≥1 error)	1.01 (0.98, 1.05)	1.00 (0.96, 1.03)	1.01 (0.98, 1.05)	1.00 (0.96, 1.03)
Sad faces (≥2 errors)	0.99 (0.95, 1.02)	1.03 (0.98, 1.07)	0.99 (0.95, 1.02)	1.03 (0.99, 1.07)
Angry faces (≥4 errors)	1.00 (0.96, 1.05)	1.00 (0.96, 1.05)	1.00 (0.96,01.04)	1.01 (0.96, 1.05)
Fearful faces (≥3 errors)	0.98 (0.94, 1.01)	0.97 (0.94, 1.01)	0.98 (0.94, 1.01)	0.97 (0.94, 1.01)
All low intensity faces (≥5 errors)	1.01 (0.98, 1.05)	1.01 (0.97, 1.04)	1.01 (0.98, 1.05)	1.01 (0.97, 1.05)
All high intensity faces (≥3 errors)	1.00 (0.97, 1.04)	0.99 (0.95, 1.03)	1.00 (0.96, 1.04)	0.99 (0.95, 1.03)
Misattributed as Happy (≥4)	0.96 (0.92, 1.01)	0.99 (0.94, 1.03)	0.96 (0.92, 1.01)	1.00 (0.95, 1.03)
Misattributed as sad (≥3)	1.01 (0.96, 1.05)	0.97 (0.93, 1.02)	1.01 (0.96, 1.05)	0.97 (0.93, 1.02)
Misattributed as Angry (≥2)	1.01 (0.96, 1.06)	1.02 (0.97, 1.06)	1.01 (0.96, 1.05)	1.02 (0.97, 1.06)
Misattributed as fearful (≥2)	1.00 (0.97, 1.04)	0.99 (0.96, 1.03)	1.00 (0.96, 1.04)	0.99 (0.96, 1.03)
Male				
Happy faces (≥1 error)	1.01 (0.97, 1.04)	0.99 (0.97, 1.00)	1.01 (0.99, 1.02)	0.99 (0.97, 1.00)
Sad faces (≥2 errors)	1.02 (0.98, 1.06)	1.02 (0.99, 1.04)	1.02 (0.99, 1.04)	1.02 (0.99, 1.04)
Angry faces (≥4 errors)	1.02 (0.99, 1.06)	1.03 (0.99, 1.07)	1.03 (0.99, 1.07)	1.03 (0.99, 1.07)
Fearful faces (≥3 errors)	1.02 (0.99, 1.07)	1.02 (0.99, 1.04)	1.02 (0.99, 1.05)	1.02 (0.99, 1.04)
All low intensity faces (≥5 errors)	1.05 (1.01, 1.09)^ns^	1.02 (0.99, 1.06)	1.05 (1.01, 1.09)^ns^	1.02 (0.99, 1.06)
All high intensity faces (≥3 errors)	1.01 (0.97, 1.05)	1.00 (0.97, 1.03)	1.01 (0.99, 1.03)	1.00 (0.97, 1.03)
Misattributed as Happy (≥4)	1.01 (0.97, 1.06)	1.01 (0.70, 1.05)	1.01 (0.97, 1.06)	1.01 (0.70, 1.05)
Misattributed as sad (≥ three)	1.01 (0.97, 1.05)	1.00 (0.97, 1.04)	1.01 (0.99, 1.03)	1.00 (0.97, 1.04)
Misattributed as angry (≥2)	1.02 (0.97, 1.07)	1.00 (0.98, 1.02)	1.02 (0.97, 1.07)	1.00 (0.98, 1.02)
Misattributed as fearful (≥2)	1.00 (0.97, 1.04)	1.00 (0.98, 1.01)	1.00 (0.97, 1.04)	1.00 (0.98, 1.01)

Higher scores in DANVA means more mistakes are made when identifying emotions/more misattributions

*ns* not significant after adjusting for multiple comparisons using the Bonferroni-Holm method

^a^Minimally adjusted model: adjusted for child age and tester

^b^Fully adjusted model: adjusted for child age, tester, maternal education, ethnic background and parity; higher scores on SCDC represent more social communication problems

**Table 3:** Logistic regression analyses of children’s 2D:4D ratios: comparison of children scores in social communication (SCDC: binary and top 10 %). Both genders together and stratified by gender

	Minimally adjusted model OR (95 % CI)^a^		Fully adjusted model OR (95 % CI)^b^	
	2D:4D right	2D:4D right	2D:4D right	2D:4D left
Binary *n* (%) scoring ≥9	1.002 (0.96, 1.05)	1.011 (0.97, 1.06)	1.003 (0.97, 1.04)	1.013 (0.98, 1.05)
SCDC scores on top 10 %	1.012 (0.98, 1.05)	1.028 (1.00–1.06)	1.012 (0.98, 1.04)	1.028 (1.00–1.06)
Female				
Binary *n* (%) scoring ≥9	1.046 (0.98, 1.12)	1.048 (0.98, 1.12)	1.048 (0.99, 1.12)	1.048 (0.98, 1.12)
Binary top 10 %	1.020 (0.97, 1.07)	1.044 (1.00, 1.10)	1.021 (0.97, 1.07)	1.043 (0.99, 1.10)
Male				
Binary *n* (%) scoring ≥9	1.005 (0.96, 1.05)	0.986 (0.93, 1.04)	0.973 (0.92, 1.03)	0.986 (0.93, 1.04)
Binary top 10 %	1.006 (0.97, 1.04)	1.013 (0.97, 1.06)	1.005 (0.99, 1.02)	1.013 (0.97, 1.06)

*ns* not significant after adjusting for multiple comparisons using the Bonferroni-Holm method

^a^Minimally adjusted model: adjusted for child age and tester

^b^Fully adjusted model: adjusted for child age, tester, maternal education, ethnic background and parity

ns: < 0.001

**Table 4:** Linear regression of children’s emotion recognition (emotional triangles) scores: both genders together and stratified by gender

	Minimally adjusted model B (95 % CI)		Fully adjusted model B (95 % CI)		R^2^(adjusted model)
	2D:4D right	2D:4D left	2D:4D right	2D:4D left	
Angry	0.010 (−0.01, 0.03)	0.001 (−0.01, 0.02)	0.010 (−0.01, 0.02)	0.002 (−0.01, 0.02)	0.01
Happy	0.019 (0.00, 0.04)^ns^	0.014 (−0.00, 0.03)	0.019 (0.0, 0.04)^ns^	0.015 (−0.00, 0.03)	0.01
Sad	0.003 (−0.01, 0.02)	−0.002 (−0.02, 0.01)	0.002 (−0.01, 0.02)	−0.003 (−0.02, 0.01)	0.01
Scared	0.007 (−0.01, 0.02)	0.007 (−0.01, 0.02)	0.007 (−0.01, 0.02)	0.007 (−0.01, 0.02)	0.03
Female					
Angry	0.007 (−0.01, 0.03)	0.005 (−0.02, 0.03)	0.006 (−0.02, 0.03)	−0.001 (−0.02, 0.02)	0.01
Happy	0.021 (−0.00, 0.04)	0.015 (−0.01, 0.04)	0.020 (0.00, 0.04)	0.015 (−0.01, 0.04)	0.01
Sad	0.004 (−0.01, 0.02)	−0.001 (−0.00, 0.00)	0.003 (−0.01, 0.02)	−0.001 (−0.02, 0.02)	0.01
Scared	−0.001 (−0.02, 0.02)	0.014 (−0.01, 0.04)	−0.002 (−0.02, 0.02)	0.000 (−0.02, 0.02)	0.02
Male					
Angry	0.007 (−0.01, 0.03)	−0.003 (−0.02, 0.02)	0.006 (−0.02, 0.03)	0.005 (−0.01, 0.02)	0.01
Happy	0.021 (−0.00, 0.04)	0.013 (−0.01, 0.04)	0.020 (0.00, 0.04)	0.014 (−0.01, 0.04)	0.01
Sad	0.004 (−0.01, 0.02)	−0.003 (−0.02, 0.02)	0.003 (−0.01, 0.02)	−0.004 (−0.02, 0.01)	0.01
Scared	−0.001 (−0.02, 0.02)	−0.001 (−0.02, 0.02)	−0.002 (−0.02, 0.02)	0.015 (−0.01, 0.04)	0.01

*ns* not significant after adjusting for multiple comparisons using the Bonferroni-Holm method

^a^Minimally adjusted model: adjusted for child age, child gender, and tester

^b^Fully adjusted model: adjusted for child age, tester, maternal education, ethnic background and parity

***p ≤ 0.001

Significant associations were found between extreme levels of 2D:4D ratios (the lowest 10 %) and scores on both DANVA and SCDC measures (see Table 5). Individuals who had the lowest 10 % of right 2D:4D ratios (i.e., the most male-biased ratio) were significantly more likely to have SCDC scores over the “probably autistic” threshold (higher than cut-off) (OR = 1.65, 95 % CI 1.1–2.4, *p* < 0.01). Those in the lowest 10 % of right 2D:4D ratios were significantly more likely to make more mistakes in recognizing emotions for sad facial expressions on the DANVA; OR = 1.49, 95 % CI 1.09–2.17, *p* < 0.05 and facial expressions of low intensity (all expressions), OR = 1.35, 95 % CI 0.98–1.79, *p* < 0.05. Individuals with the lowest 10 % of right 2D:4D ratios were also more likely to misattribute facial expressions (of whatever type) as angry; OR = 1.64, 95 % 1.16–2.33, *p* < 0.01). These odds ratios held only for males after stratifying by gender. No significant associations were found between the lowest 10 % of left 2D:4D ratios and SCDC or DANVA scores in either sex. No significant associations were found between the lowest 10 % of 2D:4D ratios (both the right and left hand) and scores in the Emotion Triangles task (see Table 6). Significant associations between 2D:4D ratios and outcome variables (SCDC and DAWBA) were also investigated using loess smoothing plots (locally weighted scatterplot smoothing) to model the change between each binary outcome and 2D:4D as a continuous variable (see Additional file 4: figures S1, S2, S3, and S4). The plots graphically show that at the lower end of the distribution in 2D:4D, the association between this variable and all outcomes changes.

**Table 5:** Logistic regression analyses of children’s 2D:4D ratios (bottom 10 %): comparison of children scoring high and low on emotion recognition (DANVA) and social communication (SCDC)

	OR (95 % CI)	OR (95 % CI)^a^	OR (95 % CI)^a^	OR (95 % CI)^a^	OR (95 % CI)^a^
2D:4D cut-off	SCDC (at 13)	DANVA (happy faces)	DANVA (sad faces)	DANVA (angry faces)	DANVA (fearful faces)
Bottom 10 % RH 2D:4D	1.65 (1.12–2.43)**	1.23 (0.93–1.61)	1.33 (0.98–1.82)****	1.19 (0.85–1.64)	1.22 (0.89–1.67)
Males	1.86 (1.18–2.93)**	1.32 (0.93–1.89)	1.49 (1.09–2.17)*	1.39 (0.94–2.04)****	1.42 (0.97–2.13)****
Females	1.32 (0.62–2.81)	0.91 (0.55–1.43)	1.02 (0.60–1.72)	0.62 (0.31–1.23)	0.90 (0.53–1.54)
Bottom 10 % LH 2D:4D	1.18 (0.76–1.83)	1.03 (0.76–1.39)	0.84 (0.59–1.18)	1.01 (0.72–1.43)	0.84 (0.60–1.19)
Males	1.28 (0.77–2.13)	0.95 (0.65–1.39)	0.75 (0.57–1.35)	1.06 (0.70–1.61)	0.94 (0.61–1.45)
Females	0.99 (0.42–2.31)	1.00 (0.60–1.64)	0.69 (0.38–1.27)	0.71 (0.36–1.39)	0.66 (0.37–1.20)
	OR (95 % CI)^a^	OR (95 % CI)^a^	OR (95 % CI)^a^	OR (95 % CI)^a^	OR (95 % CI)^a^
	DANVA (low intensity faces)	DANVA (high intensity faces)	DANVA (misattributed as happy)	DANVA (misattributed as sad)	DANVA (misattributed as angry)
Bottom 10 % RH 2D:4D	1.35 (1.01–1.82)*	1.32 (0.98–1.79)****	0.83 (0.56–1.22)	1.19 (0.85–1.67)	1.64 (1.16–2.33)**
Males	1.61 (1.14–2.33)**	1.52 (1.05–2.17)*	0.79 (0.49–1.28)	1.45 (0.98–2.13)****	2.04 (1.32–3.13)**
Females	0.84 (0.49–1.43)	0.94 (0.56–1.59)	0.81 (0.43–1.54)	0.50 (0.23–1.10)****	1.22 (0.68–2.17)
Bottom 10 % LH 2D:4D	1.05 (0.77–1.43)	0.83 (0.60–1.16)	0.67 (0.44–1.02)	0.94 (0.66–1.35)	0.96 (0.64–1.04)
Males	1.12 (0.77–1.64)	0.89 (0.59–1.35)	0.63 (0.37–1.06)	1.12 (0.75–1.69)	0.96 (0.56–1.64)
Females	0.80 (0.46-1.39)	0.65 (0.35–1.16)	0.68 (0.34–1.37)	0.36 (0.15–0.91)	0.96 (0.50–1.85)

Higher scores on SCDC represent more social communication problems. Higher scores in DANVA means more mistakes are made when identifying emotions/more misattributionss

*p ≤ 0.05; **p ≤ 0.01; ***p ≤ 0.001; ****p < 0.1

**Table 6:** Linear regression of children’s 2D:4D ratios (bottom 10 %): comparison of children’s scores in emotion recognition (emotional triangles)

	B (95 % CI)	B (95 % CI)	B (95 % CI)	B (95 % CI)
2D:4D cut-off	Emotional triangles (angry)	Emotional triangles (happy)	Emotional triangles (sad)	Emotional triangles (scared)
Bottom 10 % RH 2D:4D	−0.06 (−0.21, 0.10)	−0.01 (−0.19, 0.17)	−0.05 (−0.19, 0.09)	−0.07 (−0.23, 0.10)
Bottom 10 % LH 2D:4D	0.08 (−0.08, 0.24)	−0.01 (−0.19, 0.17)	0.08 (−0.05, 0.22)	0.02 (−0.14, 0.18)

*p ≤ 0.05; **p ≤ 0.01; ***p ≤ 0.001; ****p < 0.1

### Circulating maternal testosterone

Results are shown in Table 7. A significant negative correlation was found between left 2D:4D ratios and circulating maternal testosterone levels during pregnancy (*r* = −0.28). No significant correlation was found between the right-hand 2D:4D and maternal testosterone levels.

**Table 7:** Correlation coefficient (Spearman’s rho) of maternal testosterone levels during pregnancy (nmol/l blood) and children’s 2D:4D ratios

	R2D:4D	L2D:4D
Testosterone nmol/L blood	−0.19	−0.28*

*p ≤ 0.05; **p ≤ 0.01; ***p ≤ 0.001; ****p < 0.1

## Discussion

The aim of this study was to investigate a possible association between autistic-like social communication and emotion recognition problems and 2D:4D ratios across genders. Furthermore, we wanted to test the hypothesis that maternal circulating testosterone levels during pregnancy would be correlated with 2D:4D ratios.

Despite our relatively large general population sample, there was minimal evidence that 2D:4D ratio was associated with autistic-like traits overall. We found a modest association between the right hand digit ratio and poorer recognition of low-intensity faces; however, this difference did not withstand correction for multiple testing. In contrast, we found evidence that those with exceptionally low-right hand 2D:4D ratios had higher odds of being amongst the top 10 % on autistic traits and of making mistakes in recognizing sad facial expressions, recognition of low-intensity emotional faces and misattributions as angry. There was evidence of a non-linear relationship between DANVA and SCDC scores and the low extreme of 2D:4D. High maternal testosterone levels were correlated with the lower left hand 2D:4D ratios in a subsample, suggesting that 2D:4D ratios might be a valid proxy measure of testosterone exposure in pregnancy. However, we found no evidence of an association between maternal testosterone levels during pregnancy and child’s social communication and emotion recognition.

Our failure to find an association between 2D:4D ratios as a continuous measure and autistic traits, as measured by the SCDC and the emotion recognition measures, does not support the EMB theory of autism. This theory predicts that 2D:4D ratios in both genders correlate with autistic characteristics, such that lower ratios are associated with ASD traits, less empathy and a more systemizing cognitive stance [8]. Past studies have yielded mixed results [38, 39, 53], and have largely not been replicated; furthermore, most studies to the date have relied on relatively small samples, which limited their power to detect effects. A large community-based study by Manning and colleagues [35], the largest to date, found a significant negative correlation (males *r* = −0.013; females *r* = −0.019) between higher scores on the systemizing quotient and lower 2D:4D ratios [35]. This study is the only large community-based study conducted to date that has found a significant correlation. However, this study has methodological shortcomings, including self-measurement of the finger lengths, which has been shown to be unreliable [54] (a recent meta-analysis estimated the reliability of the measurement to be 46 % of that of expert-measured 2D:4D) [17]; reliance on a subset of not validated questions to assess systemizing, and selection bias due to the nature of the recruited sample (internet based survey) [55].

Our findings of an association between the extreme 2D:4D ratios (likely to correspond to a very high prenatal testosterone exposure), SCDC measures of autistic features and DANVA scores in males in our post hoc analyses, suggest that the association between autistic traits and testosterone exposure might occur only at the extreme and is confined to males. We did not find the same influence of fetal testosterone in females, even in those who had been subject to a relatively high exposure of early testosterone as measured indirectly by their low 2D:4D ratios. This is an interesting finding, as the EMB theory suggests that fetal testosterone has a masculinizing effect and might play a role in the development of ASD. Therefore, one might expect to find the same influence of fetal testosterone in females, with high exposure to early testosterone (low 2D:4D) having a masculinizing effect.

There are two possible explanations for our failure to replicate previous reports of an association between 2D:4D ratios and overall autistic traits and our novel findings of an association at the extremes of 2D:4D ratios in this sample:

1. That previous results suggesting fetal testosterone exposure is associated with empathizing and systemizing measures are biased. One possible hypothesis for this is a bias that the specific questions in the systemizing and empathizing measures were developed based on gender specific characteristics and this might affect assessment of these traits.
2. That a threshold effect exists, and the relationship between fetal testosterone and ASD traits is non-linear, such that relatively high levels of fetal testosterone (in the highest 10 % at least) are needed to render males more vulnerable to developing autistic-like traits.

### Strengths and limitations

This study has several strengths: unlike past studies, it is based on a large community sample which provided more power to detect weak effects than the past research on smaller samples. The use of prospective data collection and the assessment of emotion recognition using two different measurement tools are important strengths. Furthermore, the measures for this study were collected independent of the current research question, reducing or eliminating rater bias.

The ALSPAC cohort, although representative of the Avon area, is not a representative of the UK as a whole [42], and children who attended face-to-face assessments were of higher SES, with older and better educated mothers [42, 43]. ALSPAC children who were not lost to follow up are less ethnically diverse compared to the UK population. This limits the generalizability of our findings. Although we adjusted for these sociodemographic variables in the analyses, it is possible that there was some residual confounding.

The 2D:4D ratio is only a proxy measure of fetal exposure to testosterone [56], but recent reviews have demonstrated its usefulness and reliability [19, 56, 57]. Furthermore, the direct measurement of prenatal testosterone is associated with high economic and health risks; therefore, using proxy measurements that are valid are desirable. However, it is important to note that the use of finger ratios is still controversial and it has limitations. Therefore, any conclusions from the study should take the possible limitations of the measure into account. Another limitation in the study is the large variation in the time prenatal testosterone samples were collected, although 75 % were collected between gestation weeks 8–24, the period of testosterone surge in male fetuses.

A final limitation of the study relates to the nature of variables used. Some variables used were count variables (e.g., SCDC) with many zero values and could not be normalized; we therefore used recommended cut-offs, provided by the authors of the measures, which are informative at a clinical level. Dichotomizing variables however leads to the loss of potentially relevant information in the variability of measures.

## Conclusions

Prior research into the association between 2D:4D ratios and the presence of autistic-like traits have resulted in mixed findings. This study overcomes some of the limitations of previous general population studies because of the relatively large sample size, the recruitment of a mixed gender sample, and our use of a direct measure of maternal circulating testosterone during pregnancy. We found an association between an extremely low 2D:4D ratio and social communication/emotion recognition difficulties suggesting a possible threshold effect. Our positive finding was confined to males and needs replication.

## Additional files

Additional file 1: Table S1. Comparison of sociodemographic data of the ALSPAC cohort and the sample included in the current study. Additional file 2: Table S2. Sociodemographics across sample studied. Additional file 3: Table S3. Descriptive statistics of predictors and outcomes stratified by gender. Additional file 4: Figures S1–S4.
**Figure S1:** Lowess regression line for DANVA sad faces against the right hand 2D:4D. **Figure S2:** Lowess regression line for DANVA low-intensity faces against the right 2D:4D. **Figure S3:** Lowess regression line for DANVA high-intensity faces against the right 2D:4D. **Figure S4:** Lowess regression line for DANVA faces misattributed as angry against the right hand 2D:4D.

## Abbreviations

2D:4D: second to fourth digit ratio
ALSPAC: Avon Longitudinal Study of Parents and Children
AQ: autism quotient
ASD: autism spectrum disorders
DANVA: Diagnostic Analysis of Nonverbal Accuracy
EMB: extreme male brain
EQ: empathising quotient
RMET: Reading the Mind in the Eyes Test
SCDC: Social and Communication Disorders Checklist
