# Supplementary material for: Social communication and emotion difficulties and second to fourth digit ratio in a large community-based sample
Source: Mol Autism. 2015 Dec 28;6:68. doi: 10.1186/s13229-015-0063-7 (PMC4693443; doi:10.1186/s13229-015-0063-7)
Supplement: Additional file 1: Table S1. — Comparison of sociodemographic data of the ALSPAC cohort and the sample included in the current study. [file 13229_2015_63_MOESM1_ESM.docx]

**Table S1** Comparison of socio-demographic data of the ALSPAC cohort and the sample included in the current study

|  | **Whole ALSPAC Sample (N=11,868)** | **Sample available for Analysis (N=3,515)** | **Chi-square (df, N), p value** |
| --- | --- | --- | --- |
| **Child gender** (male) | 7,573 (49.7%) | 1,718 (48.9%) | 13.11(1,14694), p < 0.001 |
| **Child ethnicity** (white) | 10,715 (70.4%) | 3,326 (94.6%) | 74.87(2,11590), p < 0.001 |
| **Parity** (multiparous) | 6,473 (42.5%) | 1,741 (49.5%) | 47.99(1,11716), p < 0.001 |
| **Marital status of Mother** (married) | 9,085 (59.7%) | 2,942 (83.7%) | 164.35(1, 11868), p < 0.001 |
| **Maternal education** (up to O level) | 7,351 (48.3%) | 1,698 (48.3%) | 345.17(1,11521), p < 0.001 |

1. ALSPAC = Avon Longitudinal Study of Parents and Children
