# Supplementary material for: Social communication and emotion difficulties and second to fourth digit ratio in a large community-based sample
Source: Mol Autism. 2015 Dec 28;6:68. doi: 10.1186/s13229-015-0063-7 (PMC4693443; doi:10.1186/s13229-015-0063-7)
Supplement: Additional file 2: Table S2. — Sociodemographics across sample studied. [file 13229_2015_63_MOESM2_ESM.docx]

**Table S2**. Socio demographics across sample studied

|  | **N=3,515** |
| --- | --- |
|  |  |
| **Child gender, %** |  |
| Male | 1718 (48.9%) |
| Female | 1797 (51.1%) |
| Missing | 0 |
|  |  |
| **Marriage Stability, %** |  |
| Single | 549 (15.6%) |
| Married | 2942 (83.7%) |
| Missing | 24 (0.7%) |
|  |  |
| **Parity, %** |  |
| Primiparae | 1718 (48.9%) |
| Multiparae | 1741 (49.5%) |
| Missing | 56 (1.6%) |
|  |  |
| **Maternal education, %** |  |
| Up to O level | 1778 (50.6%) |
| A level or higher | 1698 (48.3%) |
| Missing | 39 (1.1%) |
|  |  |
| **Child ethnic background, %** |  |
| White | 3326 (94.6%) |
| Other ethnicity | 111 (3.2%) |
| Missing | 45 (1.3%) |
|  |  |
| **Maternal age, mean (SD)** | 29.51 (4.3) |
