# Supplementary material for: Social communication and emotion difficulties and second to fourth digit ratio in a large community-based sample
Source: Mol Autism. 2015 Dec 28;6:68. doi: 10.1186/s13229-015-0063-7 (PMC4693443; doi:10.1186/s13229-015-0063-7)
Supplement: Additional file 3: Table S3. — Descriptive statistics of predictors and outcomes stratified by gender. [file 13229_2015_63_MOESM3_ESM.docx]

**Table S3**. Descriptive statistics of predictors and outcomes stratified by gender

|  | **Total**  **N=3,515** | **Male**  **N=1,718** | **Female**  **N=1797** |  |  |
| --- | --- | --- | --- | --- | --- |
|  |  |  |  | **p value** | **effect size (Cramer’s V or cohen d** **, depending on the type of variable)** |
|  |  |  |  |  |  |
| **DANVA, %** |  |  |  |  |  |
| Happy faces (at least 1 error) | 807 (23%) | 459 (26.7%) | 348 (19.4%) | <0.001*** | 0.175 |
| Sad faces (at least 2 errors) | 618 (17.6%) | 334 (19.4%) | 284 (15.8%) | 0.005** | 0.100 |
| Angry faces (at least 4 errors) | 563 (16%) | 330 (19.2%) | 233 (13%) | <0.001*** | 0.167 |
| Fearful faces (at least 3 errors) | 628 (17.9%) | 310 (18%) | 318 (17.7%) | 0.788 | 0.009 |
| All faces (at least 7 errors) | 805 (22.9%) | 436 (25.4%) | 369 (20.5%) | 0.001*** | 0.115 |
| Low intensity faces (at least 5 errors) | 720 (20.5%) | 391 (22.8%) | 329 (18.3%) | 0.001*** | 0.110 |
| High intensity faces (at least 3 errors) | 701 (19.9%) | 378 (22%) | 323 (18%) | 0.003** | 0.101 |
|  |  |  |  |  |  |
| **SCDC, %** |  |  |  |  |  |
| Below 8/9 cut-off | 3303 (94.0%) | 1591 (92.6%) | 1712 (95.3%) | 0.001*** | 0.112 |
| Above 8/9 cut-off | 212 (6%) | 127 (7.4%) | 85 (4.7%) |  |  |
|  |  |  |  |  |  |
| **Emotional triangles, mean (SD)** |  |  |  |  |  |
| Angry | 2.62 (1.42) | 2.77 (1.40) | 2.47 (1.43) | <0.001*** | 0.209 |
| Happy | 2.10 (1.62) | 2.11 (1.72) | 1.09 (1.52) | 0.758 | 0.010 |
| Sad | 1.56 (1.24) | 1.51 (1.26) | 1.61 (1.22) | 0.017** | 0.081 |
| Scared | 2.16 (1.49) | 2.42 (1.46) | 1.91 (1.48) | <0.001*** | 0.351 |
|  |  |  |  |  |  |
| **2D:4D, mean (SD)** |  |  |  |  |  |
| Right 2D:4D | 0.96 (0.03) | 0.96 (0.03) | 0.97 (0.3) | <0.001*** | 0.331 |
| Left 2D:4D | 0.97 (0.03) | 0.96 (0.03) | 0.97 (0.3) | <0.001*** | 0.293 |

*p≤0.05, **p≤0.01, ***p≤0.001, †p<0.1
