# Supplementary material for: Social communication and emotion difficulties and second to fourth digit ratio in a large community-based sample
Source: Mol Autism. 2015 Dec 28;6:68. doi: 10.1186/s13229-015-0063-7 (PMC4693443; doi:10.1186/s13229-015-0063-7)
Supplement: Additional file 4: Figures S1–S4. — Figure S1: Lowess regression line for DANVA sad faces against the right hand 2D:4D. Figure S2: Lowess regression line for DANVA low-intensity faces against the right 2D:4D. Figure S3: Lowess regression line for DANVA high-intensity faces against the right 2D:4D. Figure S4: Lowess regression line for DANVA faces misattributed as angry against the right hand 2D:4D. [file 13229_2015_63_MOESM4_ESM.docx]

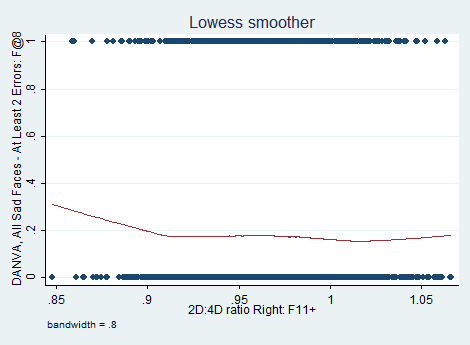
**Figure S1:** Lowess regression line for DANVA sad faces against right hand 2D:4D

**Figure S2:** Lowess regression line for DANVA low intensity faces against right 2D:4D

**Figure S3:** Lowess regression line for DANVA high intensity faces against right 2D:4D


**Figure S4:** Lowess regression line for DANVA faces misattributed as angry against right hand 2D:4D
